# Enhanced Simultaneous Nitrogen and Phosphorus Removal in A Denitrifying Biological Filter Using Waterworks Sludge Ceramsite Coupled with Iron-Carbon

**DOI:** 10.3390/ijerph16152646

**Published:** 2019-07-24

**Authors:** Xiaoying Zheng, Mengqi Jin, Hang Xu, Wei Chen, Yuan Zhang, Mengmeng Yang, Xiaoyao Shao, Zhi Xu, Weihong Wang

**Affiliations:** 1 Key Laboratory of Integrated Regulation and Resource Development on Shallow Lake of Ministry of Education, College of Environment, Hohai University, Nanjing 210098, China; 2 College of Hydraulic and Civil Engineering, Xinjiang Agricultural University, Nongda east road No.311, Sayibak District, Urumqi, Xinjiang Uygur Autonomous Region, China, 830052

**Keywords:** Waterworks sludge ceramsite, denitrifying biological filter, iron-carbon internal electrolysis, secondary effluent, nitrogen and phosphorus removal

## Abstract

In this study, waterworks sludge ceramsite (WSC) was combined with 3% iron-carbon matrix in a denitrifying biological filter (ICWSC-DNBF) to enhance the simultaneous removal of carbon, nitrogen and phosphorus in secondary effluent of wastewater treatment plant (SE-WTP). The chemical oxygen demand (COD) and nitrogen removal, as well as phosphorus removal and the adsorbed forms of phosphorus were measured and the removal mechanism of these pollutants by the ICWSC-DNBF system for treating SE-WTP were investigated. The results showed that the ICWSC-DNBF achieved good removals of COD, NH_4_^+^-N, NO_3_^−^-N, total N and total P; effluent concentrations were 17.23 mg/L, 3.72 mg/L, 14.32 mg/L, 17.38 mg/L and 0.82 mg/L, respectively. WSC enhanced the P removal due to its high specific surface area and the high number of adsorption sites. Fe-P and Al-P were the main forms of P adsorbed by WSC, accounting for 78.53% of the total adsorbed P. WSC coupled with Fe and C improved the biodegradability of SE-WTP and promoted the removal of organic matter. The removal of N was attributed to the abundant denitrifying microorganisms in the system and the electrochemical effect produced by the internal electrolysis of Fe and C.

## 1. Introduction

Recently, serious water resource problems, such as shortage of freshwater, deteriorated water quality, threats to public health by water pollution and other environmental issues, have attracted global attention. Therefore, the requirements for water resources protection and water pollution control have become increasingly stringent, and water reuse has become more important in many places. Secondary effluent from wastewater treatment plant (SE-WTP), as an unconventional water resource, has also attracted increasing attention. However, limited by sewage treatment technology, SE-WTP contains high concentrations of nitrogen (N), phosphorus (P) and other pollutants. When SE-WTP is used to recharge landscapes (i.e., as irrigation) and groundwater, its quality affects the water environment ecosystem. In addition, SE-WTP typically exhibits a low carbon-to-nitrogen ratio, is produced in large volumes, and delivers a high pollution load. Nevertheless, SE-WTP has insufficient organic matter content to meet the requirements of further treatment by traditional biological denitrification. Thus, further N and P removal is necessary to improve the reuse potential of this resource [1]. The simultaneous and efficient removal of N and P from SE-WTP poses new challenges to traditional water treatment technologies.

Many studies about the N and P removal pathways in SE-WTP have been conducted. The most commonly reported methods for treating SE-WTP include biological aerated filters, modified membrane bioreactors, electrochemical techniques and advanced oxidation methods [2,3,4]. The denitrifying biological filter (DNBF) is an effective, economical, and widely used approach for SE-WTP treatment owing to its low cost, good denitrification effect, large biomass reduction and good biofiltration performance. There are, however, some disadvantages that limit the use of the DNBF for treating SE-WTP. These include a shortage of carbon (C) for N removal, the need for a high-quality filter media, and the low operation efficiency at low temperatures. In addition, DNBFs preferentially remove N rather than P [5]. More research is necessary regarding the design, operation, and optimization of the DNBF for simultaneous N and P removal from SE-WTP.

Filter materials have been proved to be an important influence on the performance of DNBFs. Surface properties, pore structure, specific surface area and the actual materials used as filter media are important factors affecting the application performance of biosorption carriers [6]. Ceramsite, derived from nano clay, natural minerals or waste sludge, has been widely used as a carrier for adsorbents in water treatment systems due to its useful properties of high porosity, large pore diameter, sufficient mechanical strength and favorable biocompatibility [7,8]. Waterworks sludge (WS) is a by-product of the water treatment process. WS mainly consists of organic and inorganic compounds in solid, liquid and gaseous forms, and exhibits variable physical, chemical, and biological characteristics [9,10]. Importantly, WS can be converted into porous carbonaceous matter via carbonization and volatilization under high-temperature and oxygen-deficit conditions [11]. WS also contains residual coagulants such as polyaluminum chloride, aluminum sulfate and ferric chloride, which are effective in removing phosphate [12]. 

Iron (Fe) in various forms has been applied widely in biological wastewater treatment systems to improve the removal efficiency of nutrients; its changeable chemical valence can induce various physico-biochemical processes [13]. Iron-carbon micro-electrolysis also has been used widely to increase the removal of nutrients from wastewater; this ecological technology has been proved to be a highly efficient and low-cost method in the treatment of various wastewaters [14]. Yet, a comprehensive review of the available scientific literature has revealed no research that has examined the combination of WS ceramsite and an iron-carbon system in a DNBF for SE-WTP treatment.

Therefore, the objective of this study was to determine the performance of a combined WS ceramsite and iron-carbon system in a DNBF (ICWSC-DNBF) for treatment of SE-WTP. This objective was accomplished through three major areas of study: (a) assessment of chemical oxygen demand (COD) and N removal in the systems and evaluation of the variations in microbial richness and diversity in the systems; (b) evaluation of P removal and the adsorbed forms of P in the systems; and (c) identification of the pollutant removal mechanism in the ICWSC-DNBF system. An optimized strategy for the simultaneous removal of organic matter, N and P in the ICWSC-DNBF was developed based on the study results. The results also provide a realistic and science-based reference for enhancing the performance of a denitrifying biological filter to remove organics, N and P simultaneously from SE-WTP.

## 2. Experimental

### 2.1. Materials

Waterworks sludge ceramsite (WSC) was prepared using WS, bentonite and grain husk powder as the main raw materials. The preparation process consisted of crushing, screening or grinding, pelleting and burning, conducted in the following sequence. First WS, bentonite and husk powder were pulverized using a ball grinder and sieved through a #100 mesh screen. Second, WS was mixed with bentonite and husk powder in the ratio 5:3:2. Third, the mixed materials were made into pellets (Φ 5–10). Last, the pellets were placed in muffle furnace and then heated according to a specific procedure.

The characteristics of WSC were analyzed by various means. The WSC filler was characterized by specific surface area (7.75 m^2^/g), average pore diameter (8.37 nm) and pore volume (1.98 cm^2^/g). Scanning electron microscopy showed that the internal WSC had numerous crevices and pore channels of various sizes (approximately 0.5–1 mm) (Figure S1). The WSC surface was rough and porous, and the large surface area was conducive to microbial attachment. X-ray diffraction analysis showed that the main crystalline phases of WSC were SiO_2_ and Al_2_O_3_, and that other metal elements existed in amorphous form (Figure S2).

### 2.2. Experimental Apparatus and Method

Three experimental systems were set up, all of which included the DNBF. ICWSC-DNBF consisted of WSC combined with 3% iron-carbon as the matrix. WSC-DNBF consisted of WSC as the matrix. MC-DNBF consisted of marketable (i.e., commercially available) ceramsite as the matrix. The working height of the DNBF matrix was 120 cm, comprised of a 30-cm effluent area, a 90-cm filtration layer, and a 10-cm inlet area. The hydraulic retention time of each system was 60 min with a one-cycle backwash every five days. Backwashing consisted of air flushing 24.00 L/(m^2^·s) for 4 min; air flushing at 24.00 L/(m^2^·s) and water flushing at 6.00 L/(m^2^·s) for 6 min; and water flushing at 6.00 L/(m^2^·s) for 3 min. The filter was operated for 30 consecutive days and the COD, total nitrogen (TN), NH_4_^+^-N, NO_3_^−^-N, NO_2_^−^-N and total phosphorus (TP) concentrations in the influent and effluent were measured daily. 

### 2.3. Samples Analysis

The pH, dissolved oxygen (DO), water temperature, and oxidation reduction potential (ORP) were analyzed. The pH was measured using a pB-10 pH meter (Sartorius, Germany), DO was measured using a Seven2Go^TM^ (Mettler-Toledo, USA) and ORP was measured using an HI 9143 (HANNA, Italy). Ceramsite phase composition was determined using the D8-ADVANCE X-ray diffractometer (Bruker, Germany). The cross-section morphology of samples was observed using a S4800 scanning electron microscope (Hitachi, Japan). Concentrations of COD, TN, NH_4_^+^-N, NO_3_^−^-N, NO_2_^−^-N and TP were measured according to “Standard Methods for the Examination of Water and Wastewater” (APHA, 2012). All assays were performed in triplicate. Analysis of variance (ANOVA) was used to test the significance of the results and *p* < 0.05 was considered to be statistically significant. 

Bacterial communities were measured using Illumina high-throughput sequencing technology, and the analyses were conducted by Sangon Biotech (Shanghai) Co., Ltd. Operational taxonomic units (OTUs) were clustered with a 97% similarity cutoff using UPARSE software (version 7.1, http://drive5.com/uparse/, IBM Corporation, NY, USA), and chimeric sequences were identified and removed using the UCHIME algorithm (https://www.drive5.com/). The sequences were systematically classified using the RDP classifier tool (https://rdp.cme.msu.edu) and assigned to different levels. The coverage, Shannon, Chao, ACE, and Simpson indexes were generated using MOTHUR software (https://www.mothur.org/) for each DNBF sample. 

### 2.4. Phosphorous Absorption

The P adsorption test was carried out at initial P concentration of 10 mg/L, substrate dosage of 1 mg/L and pH 7. The test was performed using a shaking table at an oscillation frequency of 160 rpm. At the same time, a small amount of supernatant solution was filtered every 20 min, 40 min, 60 min, 80 min, 100 min, 120 min, 140 min, 160 min, 180 min and 200 min to determine the TP content in the solution.

The absorption capacity of P on WSC was calculated based on Equation (1):

(1) $$q_{t}=\frac{\left(c_{0}{-c}_{t}\right)V}{m}$$

where q_t_ is the absorption capacity of P (mg/g) on WSC at time t; c_0_ refers to the initial concentration of P (mg/L); c_t_ refers the P concentration at time t (mg/L); v refers to the volume of the solution (L); and m refers to the mass (g) of WSC.

### 2.5. Analysis of Adsorbed Forms of Phosphorus

The form of adsorbed phosphorus was analyzed by the leaching method. To extract physically adsorbed P, a known amount of ceramsite saturated with adsorbed P was added into a 50 mL colorimetric tube together with 30 mL NH_4_Cl solution (1 mol/L, pH 7). The solution was oscillated in the shaker for 24 h at 160 rpm. Then the solution was poured into a centrifuge tube and centrifuged for 3 min at 4000 rpm. The mixture was then filtered through a 0.45 µm filter membrane. The filtrate residue retained on the filter membrane was put aside for further treatment to determine the P content in the filtrate, which was the physically adsorbed form of P. The method for determining Fe-P, Al-P and other forms of P by the leaching method was the same as the extraction of physically adsorbed P, except that the added solutions were different. The determination of Fe-P and Al-P forms of P were leached using 20 mL of NaOH (0.1 mol/L). The determination of Ca-P and Mg-P forms of P were leached using 20 mL of HCl (0.5 mol/L).

## 3. Results and Discussion

### 3.1. COD and Nitrogen Removal in DNBF

#### 3.1.1. COD Removal

**Figure 1:**
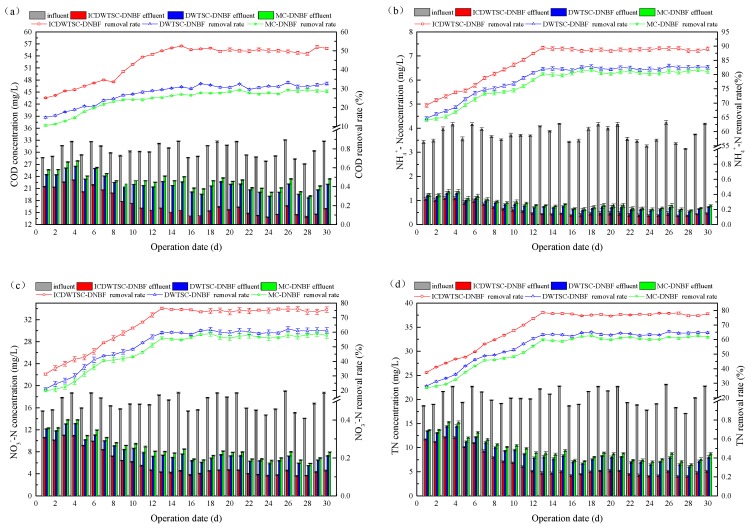
Nitrogen removal in the three systems: (**a**) COD; (**b**) NH_4_^+^-N; (**c**) NO_3_^−^-N; (**d**) TN.

Biochemical reactions occurred continuously in the three systems for 30 days. All systems received the same influent, which had a COD concentration in the range 27.09–33.09 mg/L. As shown in Figure 1a, during the 30-d study, the effluent COD concentration from the ICWSC-DNBF treatment was lower than that from both the WSC-DNBF and MC-DNBF treatments; the three systems achieved COD removal efficiencies of 51.81%, 31.20% and 28.69%, respectively. Due to the addition of iron-carbon, the average removal of COD by the ICWSC-DNBF system was 6.36 mg/L greater than that by the WSC-DNBF system. Furthermore, COD removal by the WSC-DNBF system was 4.31 mg/L greater than that in MC-DNBF due to use of WSC instead of commercial ceramsite. These results indicated that the addition of iron-carbon can improve the degradation of organics, probably because of the enhanced activity and growth rates of microorganisms [15]. WSC filler has a rich mesoporous and microporous structure, which provides more growth attachment points for microorganisms in the system than does commercial ceramsite. Thus, compared with the marketable ceramsite, the WSC was more suitable for microbial growth and microbial metabolism. 

During the first 10 d of operation, the quantity of microorganisms was limited and the biofilm on ceramsite was thin; therefore, the COD removal efficiency was low and the advantage of the ICWSC-DNBF system was not obvious. However, as the quantity of microorganisms on the ceramsite carrier increased, so did the removal of COD. After 15 d of operation, the effluent COD concentration in the three systems stabilized. Under the condition of low carbon supply, the COD removal efficiencies in the ICWSC-DNBF system were in the range of 48.52%–52.53%, and the effluent concentration was always less than 17 mg/L. This was because Fe^2+^ destroyed the carbon chains of organic contaminants effectively until they were completely mineralized to CO_2_, H_2_O and inorganic ions.

#### 3.1.2. Nitrogen Removal

The influent concentrations of TN, NH_4_^+^-N and NO_3_^−^-N were in the ranges of 17.09–23.08 mg/L, 3.43–4.24 mg/L and 14.07–18.67 mg/L, respectively. As shown in Figure 1b–d, the removal rates of TN, NH_4_^+^-N and NO_3_^−^-N in the ICWSC-DNBF system were always higher than those in the WSC-DNBF and MC-DNBF systems. During the first 10 d of operation, the removal efficiencies of these parameters fluctuated, but after 15 d of operation, the effluent TN, NH_4_^+^-N and NO_3_^−^-N concentrations in the three systems stabilized. 

During the start-up operation, NH_4_^+^-N was mainly removed by matrix adsorption, and as the number of microorganisms increased, nitrification dominated. Because the influent NH_4_^+^-N concentration was very low, the removal of NH_4_^+^-N in the three systems exceeded 80%. As reaction time increased, the effluent TN and NO_3_^−^-N concentrations from all systems decreased, while the removal rates increased and stabilized gradually. The average removal efficiencies of TN in the ICWSC-DNBF, WSC-DNBF and MC-DNBF systems were 77.38%, 63.38% and 61.29%, respectively, and the NO_3_^−^-N removal efficiencies were 75.73%, 59.42% and 56.31%, respectively.

Because nitrification mainly occurred in the biofilm, the N utilization rate was mainly related to the concentration of matrix in the biofilm, and this effect mainly depended on the external solute transfer and internal solute diffusion. The surface pores of WSC filler were relatively well developed, and the void ratio was relatively high and did not block the filter layer, so that the matrix diffusion effect and utilization rate were greatly improved (relative to that in the other systems), and the activities of ammoniated bacteria, nitrifying bacteria and nitrifying bacteria were also relatively improved [16]. Therefore, the average removal amounts of NH_4_^+^-N, NO_3_^−^-N and TN by the WSC treatment were increased 0.08 mg/L, 0.64 mg/L and 0.76 mg/L, respectively. Moreover, the results also demonstrated that due to the iron-carbon micro-electrolysis in the ICWSC-DNBF system, this system achieved better removal of N. Compared with those in the WSC-DNBF system, the average removal amounts of NH_4_^+^-N, NO_3_^−^-N and TN were increased 0.28 mg/L, 2.61 mg/L and 2.94 mg/L, respectively.

#### 3.1.3. Microbial Community Associated with COD and N Removal in DNBF

The microbial community was analyzed using high-throughput sequencing (Table 1). The Good’s coverages of all samples exceeded 99%, indicating that the microbial diversities were thoroughly described by the obtained sequence libraries. Meanwhile, the effective sequences for the ICWSC-DNBF, WSC-DNBF and MC-DNBF systems were divided into 5662, 4755, and 4533 operational taxonomic units (OTUs), respectively, based on the similarities of the domain values (0.97). The microbial richness of the iron-carbon system (ICWSC-DNBF) was higher than that of WSC-DNBF, and the microbial richness of WSC-DNBF was higher than that of MC-DNBF.

**Figure 2:**
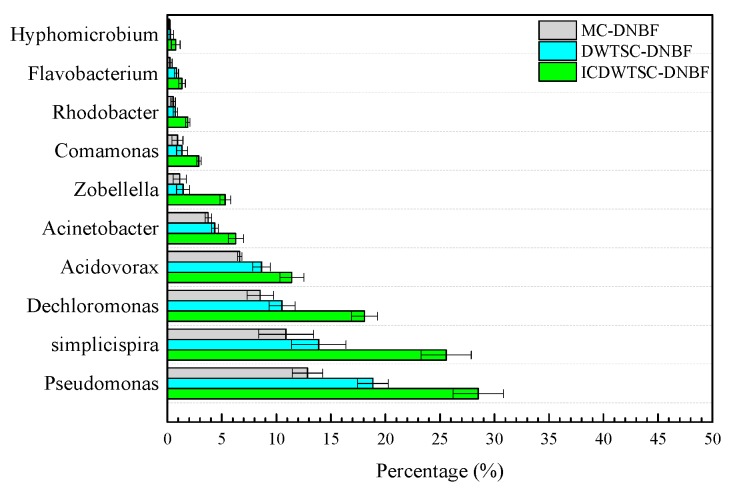
The relative abundance of the nutrient-related genera exhibiting significant differences (*p* < 0.05) in at least one of the systems.

To better characterize the differences in the functional bacteria responsible for organic matter and N removal in the three DNBF systems, the microbial communities of the DNBFs were analyzed at the genus level. The abundance of the dominant genera with significant differences (*p* < 0 .05) is presented in Figure 2. In the ICWSC-DNBF system, the average relative abundances of *Pseudomonas* (28.52%, 5.71 × 10^6^ copies/g), *Simplicispira* (25.57%, 5.12 × 10^6^ copies/g), *Dechloromonas* (18.08%, 3.36 × 10^6^ copies/g), *Acidovorax* (11.41%, 2.12 × 10^6^ copies/g), *Acinetobacter* (6.28%, 1.17 × 10^6^ copies/g), *Zobellella* (5.31%, 0.99 × 10^6^ copies/g), *Comamonas* (2.89%, 0.54 × 10^6^ copies/g), *Rhodobacter* (1.88%, 0.35 × 10^6^ copies/g), *Flavobacterium* (1.35%, 0.25 × 10^6^ copies/g) and *Hyphomicrobium* (0.78%, 0.14 × 10^6^ copies/g) were obviously higher than the corresponding abundancies in the other systems. Comparisons of microbial abundancies in the ICWSC-DNBF and WSC-DNBF systems and the corresponding N removal performances indicated that iron-carbon micro-electrolysis significantly increased the abundance of denitrifying bacteria and promoted the removal of N.

*Comamonas* is one species related to the organic matter removal that was achieved by the ten dominant genera in the three systems. *Comamonas* is a diverse microorganism. Different strains have different metabolic pathways, which play a decisive role in the degradation of phenols, quinolines and steroids. These microorganisms also play a role in the degradation of organic matter. *Simplicispira* is a special genus that can not only participate in denitrification, but also can transform some refractory organic matter. Therefore, the combination of WSC and iron-carbon prompted a much larger population of *Simplicispira* in the ICWSC-DNBF system than in the other two systems.

*Pseudomonas* was present in the largest proportion (≈29%) in all three systems, and was associated with the resistance to N removal. *Pseudomonas* is a special aerobic genus that includes a large number of aerobic and denitrifying bacteria capable of denitrification under aerobic conditions [17,18]. *Dechloromonas* are glycogen accumulating organisms that have a strong NO_3_^−^-N reduction capacity [19]. Some studies have shown that glycogen accumulating organisms can produce N_2_O during denitrification, thereby promoting N removal [20,21]. *Pseudomonas* and *Acinetobacter* have been shown to perform both heterotrophic nitrification and aerobic denitrification [22].

*Acidovorax* and *Acinetobacter, Pseudomonas, Rhodobacter* and *Hyphomicrobium* are common heterotrophic denitrifying bacteria that can utilize and directly convert organic N into NO_3_^−^-N instead of NH_4_^+^-N [23]. These microorganisms are highly efficient heterotrophic nitrifying bacteria, and have an environmental growth rate that is much higher than that of autotrophic nitrifying bacteria [24]. At the same time, these microorganisms can also participate in aerobic denitrification, thus preventing the accumulation of NO_2_^−^-N and promoting denitrification [25]. Furthermore, these bacteria are also capable of aerobic denitrification, which prevents the accumulation of both NO_2_^−^-N and NO_3_^−^-N in systems that achieve extremely high TN removal rates. 

Moreover, other heterotrophic nitrifying-aerobic denitrifying bacteria (*Zobellella*) were successfully cultivated and enriched in the DNBFs (and especially in the ICWSC-DNBF system). These bacteria played an important role in overcoming the inhibiting effects on the denitrification capacity of the systems caused by salinity and insufficient organic carbon sources [26]. Recent research has shown that *Zobellella* has a comprehensive denitrification ability, achieving nearly 100% NO_2_^−^-N removal by using NO_2_^−^-N as the main electron acceptor [27]. In contrast, *Flavobacterium* was the only genus of anaerobic denitrification bacteria detected, and its relative abundance in the three systems was very low; this indicated that the denitrification in the DNBF system depended on aerobic denitrification rather than traditional anaerobic denitrification. 

The transformation of N in the DNBF included substrate adsorption, electrochemical reactions and microbial nitrification-denitrification. Among these, microbial nitrification-denitrification was considered as the main process for permanent N removal. The process of nitrification in the DNBF system has been reported to rely not on autotrophic nitrifiers (ammonia-oxidizing bacteria and nitrite-oxidizing bacteria), but instead on heterotrophic nitrifiers such as *Acinetobacter* and *Pseudomonas*. It was noteworthy that in the ICWSC-DNBF system, the abundancies of these microorganisms were all significantly higher than those in the other two systems. These results indicated that iron-carbon added to the DNFB system not only increased the abundance of bacteria, but also increased bacterial activity. 

### 3.2. Phosphorus Removal and the Adsorbed Forms of Phosphorus in DNBF

#### 3.2.1. Phosphorous Absorption Efficiency of Waterworks Sludge Ceramsite

**Figure 3:**
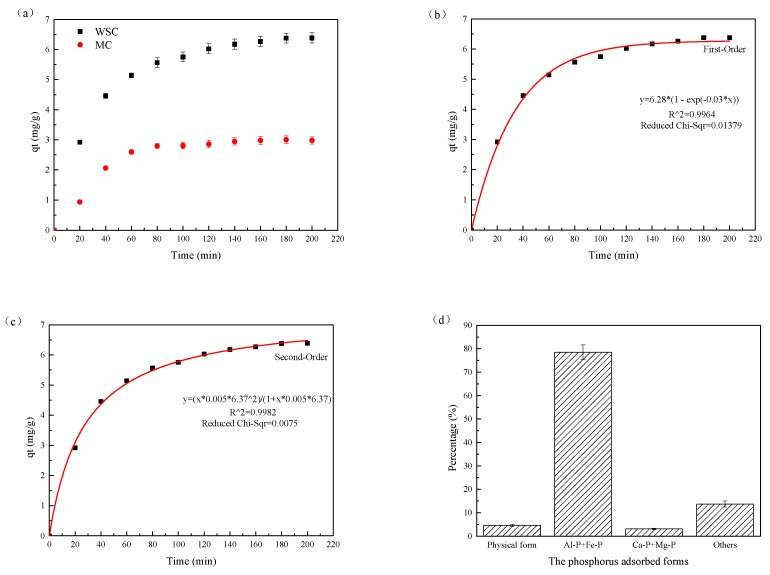
(**a**) P absorption efficiency of waterworks sludge ceramsite; (**b**) Pseudo-first-order model describing P adsorption; (**c**) Pseudo-second-order model describing P adsorption; (**d**) Adsorbed forms of P by waterworks sludge ceramsite.

The absorption of P on WSC and marketable ceramsite was examined at pH 7. As presented in Figure 3a, the adsorptive capacity of the matrix initially showed a linear, upward trend. At the beginning of the experiment, the TP concentration was high and the adsorption capacity of the matrix surface was large. Therefore, the P diffused quickly in the matrix and was adsorbed on the surface of the matrix. As reaction time increased, the amount of adsorption stabilized and approached equilibrium. The direct reasons of this problem are that the P concentration decreased in the reactor, and adsorption became the limitation to removal, so the diffusion rate was basically unchanged. After 120 min, the adsorption of P by WSC reached equilibrium (6.21 mg/g), while the adsorption by marketable ceramsite was 2.84 mg/g. In addition, the P adsorption capacity and rate of P adsorption on WSC were both higher than those on marketable ceramsite. This is attributed to the high specific surface area and the number of adsorption sites of WSC. In addition, the good precipitation and removal performance of phosphate by residual coagulants in sludge raw materials also indirectly promoted P adsorption.

To further study the P adsorbing mechanism of WSC, two kinetic models were used to describe the adsorption process: a pseudo first-order model (Equation (2)) and a pseudo second-order model (Equation (3)). The pseudo-first-order model assumed that the adsorption rate varied with the number of unoccupied sites on the matrix [28]. The pseudo second-order model was based on chemical reactions and was affected by the mass balance and the second-order rate derivative [29]. The equations were:

(2) $$\ln(q_{e}-q_{t})=\ln q_{e}-k_{1}t$$

(3) $$\frac{t}{q_{t}}=\frac{1}{k_{2}{q_{e}}^{2}}+\frac{t}{q_{e}}$$

where *q_e_* and *q_t_* are the adsorption capacities at equilibrium time and at time *t*, respectively. *k*_1_ and *k*_2_ are the rate constants of the pseudo-first-order and pseudo-secondary models, respectively.

The experimental data described using the two models are shown in Figure 3b,c. The kinetic parameters for the two models are listed in Table 2. Both models described the kinetics of WSC adsorption of P well (R^2^ 0.996–0.998), but the pseudo second-order model was marginally superior. In addition, the adsorption capacity predicted by the second-order model was 6.37 mg/g, which was close to the experimental observation (6.38 mg/g). Therefore, the adsorption of P by WSC was judged to be better described by the pseudo second-order model, and was classified as chemical adsorption.

#### 3.2.2. Adsorbed Forms of P by Waterworks Sludge Ceramsite

The step-by-step leaching analysis revealed the phosphorus adsorption morphology of WSC and the total P extracted during each step. The relative proportions of physically adsorbed P, Fe-P and Al-P, Ca-P and Mg-P are shown in Figure 3d. Fe-P and Al-P were the main forms of P adsorbed by the WSC and accounted for 78.53% of all adsorbed P. Physically adsorbed P and Ca-P and Mg-P accounted for only a small proportion of the adsorbed P, while other forms of adsorbed P accounted for 13.74%.

Waterworks sludge contains a large amount of alumina (A1_2_O_3_). Aluminum ions on the surface of alumina can interact with water molecules to form an oxide. Proton migration then occurs on the surface of the oxide to form hydroxyl groups, while the surface of hydroxyl oxide can adsorb H^+^ or OH^−^. Consequently, P can be removed by electrostatic adsorption on the surface of WSC [30]. It is noteworthy that “other” forms of adsorbed P included P precipitates that were caused by Al^3+^ and Fe^2+^ in the ceramsite. Nevertheless, the main forms and mechanism of P adsorption by WSC were Fe-P and Al-P by chemical adsorption.

#### 3.2.3. Phosphorus Removal in DNBF

**Figure 4:**
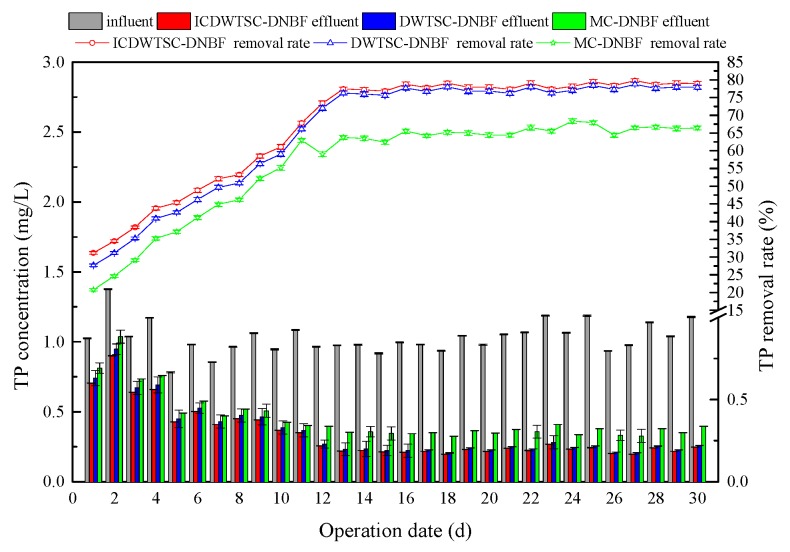
The P removed in three systems.

The influent concentrations of TP concentrations was 0.85–1.38 mg/L. During the first 10 days of operation, the TP removal efficiencies fluctuated (Figure 4). After 15 d of operation, the effluent TP concentration in the three systems stabilized. The average removal efficiencies of TP in ICWSC-DNBF, WSC-DNBF and MC-DNBF were 78.69%, 77.31% and 65.35%, respectively. The removal rate of TP in ICWSC-DNBF and WSC-DNBF was always higher than that in MC-DNBF. It is also noteworthy that there was little difference in TP removals by the ICWSC-DNBF and WSC-DNBF systems. This was because the removal of TP mainly depended on matrix adsorption and was not affected by iron-carbon. The specific surface area and the number of adsorption sites of WSC were higher than those of marketable ceramsite. In addition, the residual coagulants in sludge raw material had a good capacity for precipitation and removal of phosphate; therefore, the adsorption performance of WSC was slightly higher than that of marketable ceramsite, and the ICWSC-DNBF and WSC-DNBF systems achieved better P removal efficiency than MC-DNBF. Moreover, the results also demonstrated that due to the iron-carbon micro-electrolysis in ICWSC-DNBF systems, the ICWSC-DNBF system achieved slightly better removal of TP than did the WSC-DNBF system. The iron ion and phosphate produced by the electrochemical reaction precipitated, which improved the removal rate of TP in the system. Compared with that in the MC-DNBF system, the average removal amount of TP was 0.12 mg/L greater in the WSC-DNBF system and 0.14 mg/L greater in the ICWSC-DNBF. This performance was much better than the P removal achieved in the research of Zhi et al. [4].

### 3.3. Apparent Removal Mechanism of the ICWSC-DNBF

In the ICWSC-DNBF system, organic matter could be removed through physical adsorption onto the matrix and through metabolic decomposition by microorganisms (the predominant pathway). However, degradation of SE-WTP was difficult because its organic composition was mostly refractory macromolecules (such as humic acid, surfactants, synthetic agents, etc.) [31]. Due to the addition of iron-carbon, iron-carbon internal electrolysis occurred in the ICWSC-DNBF system. Simultaneously, the reducing or oxidizing effect of Fe^2+^ generated at the anode, together with freed electrons, effectively destroyed the carbon chains in organic contaminants until they were completely mineralized to CO_2_, H_2_O and inorganic ions [32]. Moreover, in the ICWSC-DNBF, the refractory organics (including N-containing compounds), acted as electron acceptors and were reduced to short-chain organic molecules and NH_4_^+^-N, which improved the biodegradability of SE-WTP. Furthermore, during the Fe-C micro-electrolysis oxidation or reduction process, the refractory macromolecular substances were oxidized to small intermediates or by-products, which were removed by co-precipitation and flocculation of the ferrous hydroxide and ferric hydroxide floc sludge.

The transformation of N in the DNBF included substrate adsorption, electrochemical reactions and microbial nitrification-denitrification. In general, NH_4_^+^-N was oxidized to NO_2_^−^-N and NO_3_^−^-N by the nitrifying bacteria in the system. NO_3_^−^-N was converted to N_2_ by denitrifying bacteria under anaerobic conditions. According to the microbial community analysis, the species of denitrifying bacteria were diverse and facilitated different pathways of biological denitrification. These results indicated that iron-carbon added to the system can not only increase the abundance of bacteria, but also increase bacterial activity. Moreover, WSC had a very mesoporous and microporous structure, which as the main filler in the DNBF system provided abundant attachment points for the growth of microorganisms in the system. Thus, WSC was more suitable for the growth and metabolism of microorganisms than marketable ceramsite. 

Iron-carbon also played a great role in promoting N removal from SE-WTP. Due to the iron-carbon micro-electrolysis in the ICWSC-DNBF system, the refractory organic compounds could be transformed to biodegradable organic compounds. In addition, the degraded organics also provided additional carbon for denitrification and enhanced the removal of N. Besides, NO_3_^−^-N (the main from of N in SE-WTP) could react with electrons lost by Fe (Equations (4) and (5)), which may have been an important pathway for N removal. Meanwhile, the generated ferric iron was reduced through respiration (as the electron acceptor) as well as fermentation (as an electron sink) under anaerobic conditions, and was also involved in the oxidation of NH_4_^+^-N and the reduction of NO_3_^—^N; therefore, it improved the N removal efficiency. Additionally, NH_4_^+^ could couple with Fe^3+^, and be oxidized to N_2_, NO_2_^−^-N or NO_3_^—^N under anaerobic conditions [33,34].

Fe − 2e → Fe^2+^-e → Fe^3+^ (4)

NO_3_^−^ + 5e + 3H_2_O → (1/2)N_2_ + 6OH^−^ (5)

The removal of P in the system was mainly achieved by adsorption and chemical precipitation. The adsorption of P by WSC was the dominant pathway. The large specific surface area, abundant porosity and large number of adsorption sites of WSC were beneficial to P removal [35]. Moreover, the WSC mainly contained A1_2_O_3_ and SiO_2_; thus, ion exchange and electrostatic adsorption in the system greatly improved the P removal efficiency of the ICWSC-DNBF system. Moreover, ferrous iron lost electrons to form ferric iron by oxidation in a fully aerobic condition. Then, the oxidation and precipitation of ferrous and ferric ions formed the ferrous and ferric hydroxides (such as Fe(OH)^2+^, Fe(OH)_2_^+^, etc.), which were favorable flocculants for removing both degradation-resistant organic compounds and P.

**Figure 5:**
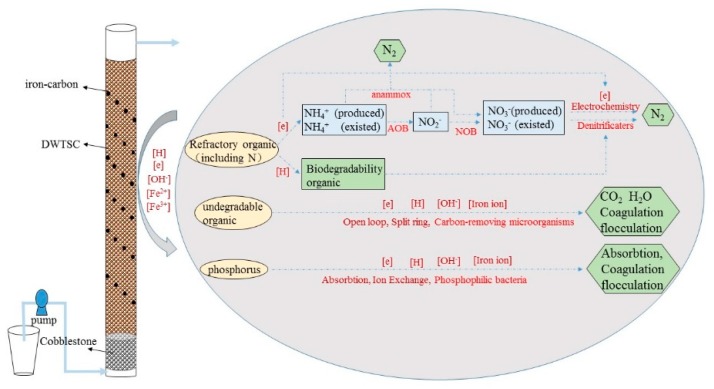
Proposed removal mechanism of organic compounds, nitrogen and phosphorus in the ICWSC-DNBF.

In general, the adsorption of P by WSC in the ICWSC-DNBF system was the main pathway of P removal. WSC coupled with iron and carbon improved the biodegradability of SE-WTP and promoted the removal of organic matter. The removal of N was attributed to the abundant denitrifying microorganisms in the system and the electrochemical effect produced by the internal electrolysis of iron and carbon. In short, many synergistic effects associated with the combination of WSC with iron-carbon can effectively degrade the organic compounds, N, and P in SE-WTP (Figure 5).

## 4. Conclusions

This research demonstrated that the ICWSC-DNBF system effectively removes C, N and P simultaneously from SE-WTP. Moreover, the iron-carbon micro-electrolysis in the ICWSC-DNBF system promotes better removal of N compared to that by the WSC-DNBF system. The use of iron-carbon enhances microbial abundance and microbial diversity in the ICWSC-DNBF system. In general, the adsorption of P by WSC is the main pathway of P removal in the ICWSC-DNBF system. WSC coupled with iron and carbon improves the biodegradability of SE-WTP and promotes the removal of organic matter. The abundant denitrifying microorganisms in the ICWSC-DNBF system and the electrochemical effect produced by the internal electrolysis of iron and carbon are mainly responsible for N removal. These study results prove the viability of a new technique (i.e., ICWSC-DNBF) for enhancing the removal of organic compounds, N and P simultaneously from SE-WTP.

## Figures and Tables

**Table 1:** Similarity-based OTUs and species richness and diversity estimates for microbial communities in the denitrifying biofilter (DNBF).

System	OUTs ^a^	Ace ^b^	Chao ^b^	Shannon ^c^	Simpson ^c^	Coverage ^d^
ICWSC-DNBF	5662	33,456.85	19,460.16	6.62	9.35 × 10^−2^	0.999
WSC-DNBF	4755	20,907.98	13,873.70	5.16	1.06 × 10^−3^	0.999
MC-DNBF	4533	26,257.87	12,990.50	5.01	1.01 × 10^−3^	0.999

^a^ OTUs: Operational taxonomic units. ^b^ Chao/Ace diversity estimator: Total amount of OTUs estimated by infinite sampling. A higher number reflects more diversity. ^c^ Shannon/Simpson richness index: Index to characterize species richness. A higher number represents higher richness. ^d^ Coverage: Estimates the possibility that the next read will belong to a specific OTU.

**Table 2:** Model-predicted kinetic parameters for P adsorption by waterworks sludge ceramsite (WSC).

q_e_, Measured (mg/g)	First-Order Model			Second-Order Model		
	k_1_	q_e_, Predicted (mg/g)	R^2^	k_2_	q_e_, Predicted (mg/g)	R^2^
6.38	0.03	6.28	0.996	0.004	6.37	0.998

## Supplementary Materials

The following are available online at https://www.mdpi.com/1660-4601/16/15/2646/s1, Figure S1: SEM picture of drinking-water treatment sludge ceramsite, Figure S2: X-ray diffraction pattern of drinking-water treatment sludge ceramsite.
