# Supplementary material for: Enhanced Simultaneous Nitrogen and Phosphorus Removal in A Denitrifying Biological Filter Using Waterworks Sludge Ceramsite Coupled with Iron-Carbon
Source: Int J Environ Res Public Health. 2019 Jul 24;16(15):2646. doi: 10.3390/ijerph16152646 (PMC6695854; doi:10.3390/ijerph16152646)
Supplement: Supplementary file 1 [file ijerph-16-02646-s001.pdf]

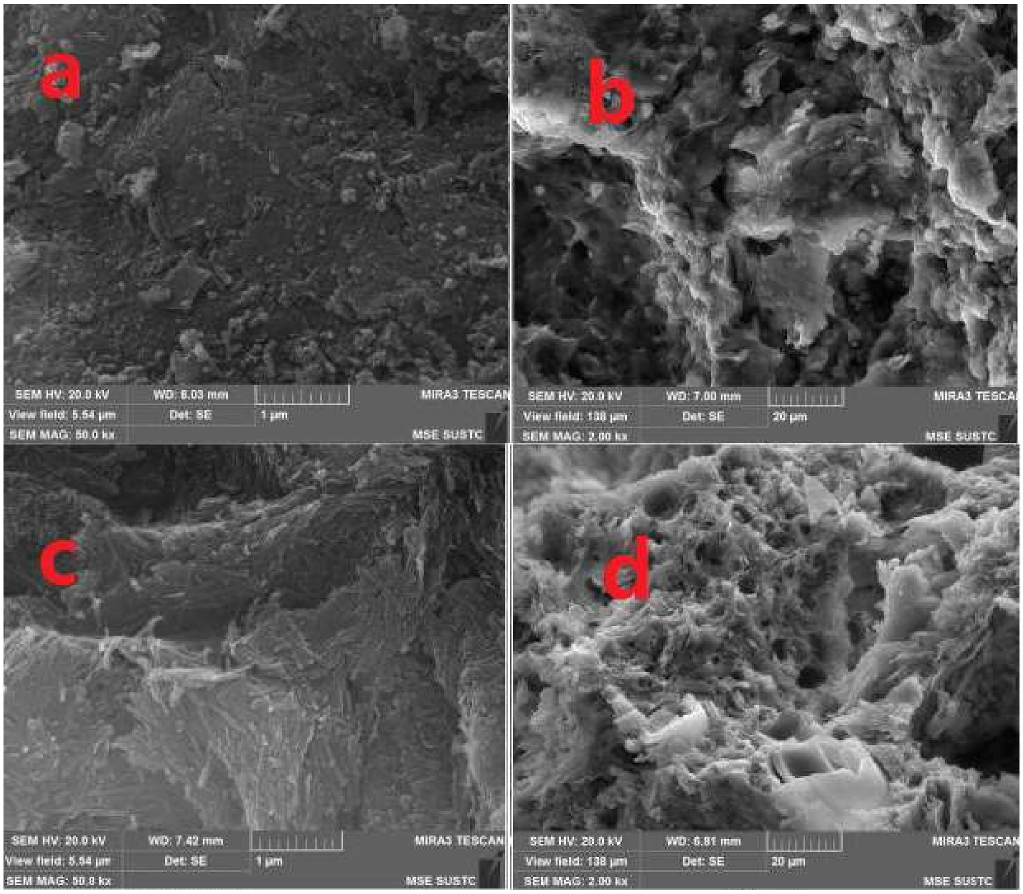

Figure S1. SEM picture of drinking-water treatment sludge ceramsite.

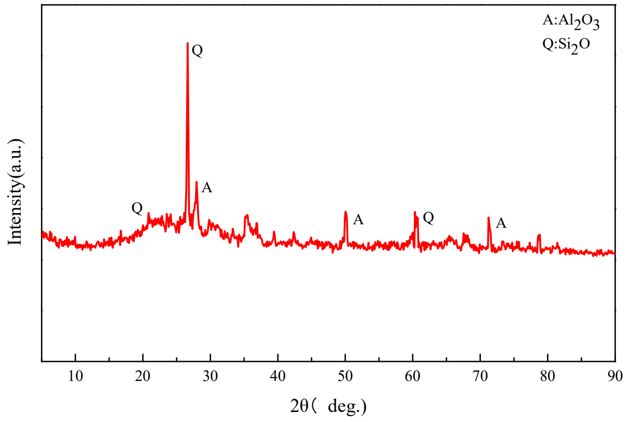

Figure S2. X-ray diffraction pattern of drinking-water treatment sludge ceramsite.
